# Measuring populations to improve vaccination coverage

**DOI:** 10.1038/srep34541

**Published:** 2016-10-05

**Authors:** Nita Bharti, Ali Djibo, Andrew J. Tatem, Bryan T. Grenfell, Matthew J. Ferrari

**Affiliations:** 1 Biology Department; Center for Infectious Disease Dynamics, Pennsylvania State University, University Park PA, USA; 2 Woods Institute for the Environment, Stanford University, Stanford CA, USA; 3 Université de Niamey, Niamey, Niger; 4 Fogarty International Center, National Institutes of Health, Bethesda, MD, USA; 5 WorldPop, Department of Geography, University of Southampton, Southampton, UK; 6 Flowminder Foundation, Stockholm, Sweden; 7 Department of Ecology and Evolutionary Biology; Woodrow Wilson School of Public and International Affairs, Princeton University, Princeton NJ, USA; 8 Department of Statistics, Pennsylvania State University, University Park PA, USA.

## Abstract

In low-income settings, vaccination campaigns supplement routine immunization but often fail to achieve coverage goals due to uncertainty about target population size and distribution. Accurate, updated estimates of target populations are rare but critical; short-term fluctuations can greatly impact population size and susceptibility. We use satellite imagery to quantify population fluctuations and the coverage achieved by a measles outbreak response vaccination campaign in urban Niger and compare campaign estimates to measurements from a post-campaign survey. Vaccine coverage was overestimated because the campaign underestimated resident numbers and seasonal migration further increased the target population. We combine satellite-derived measurements of fluctuations in population distribution with high-resolution measles case reports to develop a dynamic model that illustrates the potential improvement in vaccination campaign coverage if planners account for predictable population fluctuations. Satellite imagery can improve retrospective estimates of vaccination campaign impact and future campaign planning by synchronizing interventions with predictable population fluxes.

Infectious diseases caused an estimated 6.5 million deaths worldwide in 2010, accounting for nearly 13% of all global deaths^1^. Since 1990, impressive progress has been made in reducing disease-induced mortality, particularly a 32% decrease in common infectious diseases and an 80% reduction in measles with a widely available, low cost vaccine^1^,^2^. Unfortunately, communicable diseases still cause far too many preventable deaths; measles causes over 300,000 cases each year and nearly 140,000 deaths worldwide^3^.

Sufficient vaccination coverage has led to the local elimination of measles, and many other infectious pathogens, throughout regions of the globe^4^ but maintaining high coverage has been a challenge. Declines in vaccine coverage have lead to the re-emergence of diseases in areas where they had been previously eliminated, particularly in migrating populations^5^,^6^.

In a 1991 measles outbreak in Niamey, Niger, seasonal migration was a high risk factor for children not receiving measles immunization^7^. Similarly, migrants were more likely than non-migrants to be infected with measles during a 1997 outbreak in Sao Paolo, Brazil that resulted in over 20,000 confirmed cases^8^. In 2009, Burkina Faso experienced a measles outbreak that resulted in 54,111 cases, despite high estimated administrative vaccine coverage and several supplemental campaigns; many of the children who missed vaccinations were not locally present during campaigns and contributed significantly to the outbreak^9^. Burkina Faso’s vaccine coverage estimates preceding this outbreak suggested the country was near elimination, not at risk for a large outbreak^9^. These examples highlight the link between migration, measles transmission, and missed immunizations and can be found across many infectious diseases^10^.

Today, the majority of global measles morbidity and mortality occurs in Africa and India^2^,^3^ with recent resurgences in parts of the Middle East and areas affected by conflict due to mass displacement or the disruption of routine immunization services^11^. Measles, and many other communicable and vaccine preventable infections, disproportionately impact children, lower economic classes, and those lacking access to health care and immunizations.

The proportion of the population that needs to be immunized to interrupt disease transmission scales with the transmissibility of the disease; the highly transmissible measles virus requires that population immunity remain above 95% for local elimination^12^,^13^, emphasizing the importance of accurate population size estimates. Population estimates are most useful when they are current, updated in response to rapid or short-term movements, due to conflict, natural disasters, etc., which can prompt both outbreaks and immunization campaigns. Unfortunately, due to the scarcity of these kinds of data, population sizes are often estimated from the most recent census using linear growth rates.

## Estimating the Dynamics of Population Size

Methods to measure human populations include decadal censuses, micro-censuses, and surveys, including repeated data collection like the Demographic Health Surveys. These measurements may be temporally sparse or spatially aggregated across regional or national scales. Population estimates and projections can vary greatly between sources even when similar methods are used, which can yield dramatic discrepancies when calculating important health metrics and interventions (see Table 2 in ref. 14).

High-resolution spatiotemporal population data can be particularly difficult to access for low-income areas due to methodological insufficiencies^9^,^15^,^16^,^17^. Subnational estimates can include these areas but are often outdated and aggregated, masking important heterogeneities across socioeconomics and population characteristics^14^. Further, copyright and ownership issues can limit accessibility, even for data that are collected in national censuses.

Integrating across data sources can improve population estimates in accuracy and spatio-temporal resolution. This approach can include data on land use, land cover, or environmental conditions to capture the intrinsic relationship between human presence and changing environmental conditions (see below and refs 18 and 19). Some examples are the Gridded Population of the World (GPW)^20^, the Global Rural Urban Mapping Project (GRUMP)^21^, LandScan^22^, and WorldPop^23^ (www.worldpop.org). These products can include population characteristics for understanding health and disease, such as age structure or health care accessibility, but do not include measurements of high spatiotemporal resolution movement and migration. Recently established flowminder.org uses mobile phone data to measure movement, but access to phone data is highly restricted. Further, such recently introduced technology can’t elucidate long-term movement patterns and doesn’t provide information for areas without network coverage.

## Migration and Measles Transmission

**Figure 1:**
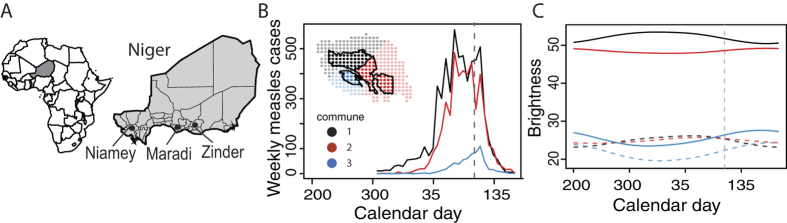
Niamey 2003–2004 brightness and measles cases. (**A**) Left: map of Africa, Niger shaded grey; right: map of Niger showing three largest cities, each is a health district. (**B**) Reported measles cases by commune for Niamey’s 2003–2004 outbreak. Vertical dashed line indicates start of reactive immunization campaign. Inset: Pixels of Niamey: communes outlined in black, faded pixels outside communes show peripheral areas. Colors indicate communes for (**B,C**). (**C**) Solid lines: brightness within each commune’s boundaries (bright pixels in (**B**)). Dashed lines: brightness for each commune including associated peripheries (bright and faded pixels in (**B**)). Vertical line: start of reactive immunization campaign. Maps are GADM shapefiles^33^ drawn and edited in R version 1.7.1 (https://cran.r-project.org)^47^, finished in Adobe Illustrator CS3 (http://www.adobe.com/products/illustrator.html).

Daily, serial satellite images of nighttime lights provide a direct, quantifiable indicator of human presence^24^,^25^ and measure intra-annual population fluctuations^17^. This approach was used to quantify seasonal population fluctuations in urban areas of Niger (Fig. 1)^17^, where large, mobile agricultural workforces migrate to urban areas during the dry season for labor opportunities (Fig. 1C)^26^. These measurements established a significant, positive correlation between measles transmission and fluctuations in population size for the three largest cities in Niger, showing that seasonal changes in population size drove local measles dynamics both across cities and within them (Figs 1A and S1)^17^,^27^,^28^.

## Outbreak and Response

Niamey experienced a large measles outbreak across all three of its communes during the 2003–2004 dry season, resulting in a total of 10,880 cases and 397 deaths (Fig. 1B)^29^. The outbreak began in communes 1 and 2, where 90% of the cases occurred. Twenty-three weeks after the start of the outbreak, an outbreak response vaccination campaign was conducted to mitigate the ongoing measles outbreak by immunizing 50% of all children younger than 60 months (Fig. 1B). The campaign ended quickly after achieving an estimated 57% coverage^29^.

Immediately following the vaccination campaign, a household survey was conducted, assessed by Lot Quality Assurance Sampling (LQAS), to measure the level of coverage achieved by the vaccination reinforcement campaign. The survey measured that the campaign achieved closer to 50.5% coverage, not the estimated of 57%. We found that this was likely due to underestimated target population sizes. The survey also found that the campaign had achieved variable coverage between the quartiers that make up the communes of the city and had not achieved the goal of 50% coverage across all areas of the city^29^. At the commune- and quartier-level within Niamey, we illustrate a link between movement and the coverage achieved by the immunization campaign. We use satellite-derived measures of seasonal population sizes and a dynamic disease model to refine our estimates of the coverage achieved by the immunization campaign. Finally, we assess alternative strategies to increase coverage for future vaccination campaigns. The methods outlined here can also be used to retrospectively improve prior vaccination coverage estimates and update future campaign goals. This approach is applicable to a variety of locations for a number of public health interventions.

## Materials and Methods

### Demographic data

We obtained estimates for Niamey’s 2004 population size from each of the following sources: *Médecins Sans Frontieres* (*MSF)*, The Ministry of Health (MoH) of Niger, and the United Nations (UN), as summarized in Table 1. All three estimates were projections from the 2001 census.

Note that *MSF* used a 2001 population size of 707951^30^ with an annual growth rate of 4.8%, calculating a total population size of 769454; our calculations using the described methodology yielded a larger total. The UN cites a 2001 census population size of 727000 for Niamey, which was higher than the census reports we identified.

The Ministry of Health provided annual estimates for the population sizes and birth rates for all the health districts in Niger for 2004, including Niamey and its communes, Maradi and Zinder (details in ref. 31).

### Cities and boundaries

In 2004, Niamey, the capital of Niger, was made up of three communes (Fig. 1B, inset map), which were further divided into quartiers (Fig. 1D, inset map). Commune 1 (19 quartiers) and commune 2 (18 quartiers) were largest in area and population size with a shared border, while commune 3 (9 quartiers) was smaller and located across the Niger River.

To map the cities and communes, we derived urban polygons and settlement polygons from 2000 and 2005 Landsat imagery from on-screen manual delineation or area-masked unsupervised per-pixel classifications sourced from the EarthSat/MDA GeoCover 2000 Stock (Landsat) scenes (as in ref. 17). We identified the pixels in the maximum lighting extent from stably lit areas, indicating permanent settlements and urban areas, through the Global Rural Urban Mapping Project (GRUMP) urban extents dataset, which is based on multiyear composite Defense Meteorological Satellite Program (DMSP)–Operational Linescan System (OLS) data^21^. These extents were used to define pixels for nighttime lights analyses using the methods described below and in ref. 17. All three communes of Niamey had populations living in the peripheral areas outside the formal commune boundaries and measles cases from these areas are reported to the city’s health care centers (Fig. 1B inset map), so no pixels were eliminated due to ‘overglow’ effect^32^.

*MSF* and the Ministry of Health provided data to generate maps of the three communes and 46 quartiers of Niamey that were used to plan and execute the vaccination campaign and subsequent household survey^29^ showed strong agreement with GIS data from Global Administrative Areas (GADM, www.gadm.org)^33^. The boundaries of each quartier were applied to image analysis to match the survey’s spatial coverage (Fig. 1B, inset map).

**Figure 2:**
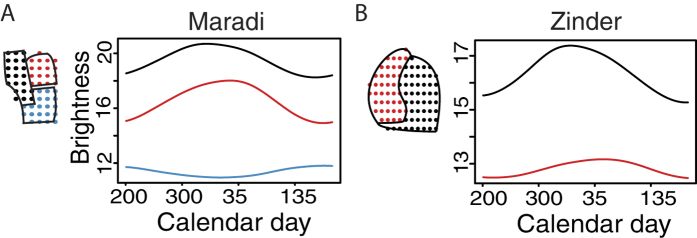
Population fluctuations within cities. (**A**) Annual brightness signature for each commune of Maradi, corresponding to pixels shown on city map at left. (**B**) Annual brightness signature for each commune of Zinder, corresponding to pixels on city map at right.

Similar to the communes of Niamey, the city of Maradi is divided into three areas, which we refer to as communes here. *MSF* provided the formal boundaries of the communes of Maradi and the divisions aligned with the major roads from GADM (Fig. 2A). Zinder is a smaller city, which we divided into two ‘communes’ on either side of a major road for this study, but they are not recognized administrative areas (Fig. 2B).

### Outbreak response vaccination campaign

In April of 2004, 23 weeks into the outbreak, MoH, WHO, and *MSF* conducted an outbreak response vaccination campaign to mitigate the outbreak by immunizing 50% of all children aged 6–59 months in the city (Fig. 1B). They distributed 84,563 vaccine doses and estimated an achieved 57% coverage in less than two weeks^29^. The number of doses administered in each commune or quartier is not known, which is not unusual for an outbreak response effort.

### Details of Lot Quality Assurance Survey (LQAS)

Three days after the conclusion of the reinforcement immunization activities, *MSF* randomly selected 65 households in each of the 46 quartiers of Niamey with at least one child between 6–59 months of age for their survey (n = 2990). Among other data, they collected each child’s history on the following: child’s date of birth, sex, history of measles infection, measles vaccination status before and during the reinforcement activities, date(s) the child received vaccination(s), and whether the information was verified with a vaccine card or provided by guardian recall. Vaccine cards are issued for all routine immunizations, so both negative and positive responses can be card-verified. Full details of the LQAS undertaken during the spring of 2004 in the quartiers of Niamey are in Dubray *et al*.^29^.

### Evaluating the immunization campaign

We evaluated commune-level differences in the proportion of children vaccinated during the campaign using a Tukey multiple comparison test across all pairwise comparisons. We calculated these commune-level results and measures of significance first using card-verified results only and then also by combining all responses (card-verified + recall).

To assess whether the immunization campaign preferentially reached unvaccinated or vaccinated children in each quartier, we performed an odds ratio test, dividing the 65 surveyed children of each quartier into the four possible combinations of vaccination history consisting of (1) previously unvaccinated/unvaccinated during reinforcement campaign, (2) previously vaccinated/ unvaccinated during reinforcement campaign, (3) previously unvaccinated/vaccinated during reinforcement campaign, and (4) previously vaccinated/vaccinated during reinforcement campaign. We completed this analysis using only children with vaccine cards. We compare those results to a parallel analysis that included information from vaccine cards and maternal recall.

### Nighttime lights data

These methods are fully explained in ref. 17, relevant details are included below.

DMSP satellites orbit at an altitude of 833 km and provide simultaneously captured pairs of visible and thermal-infrared (TIR) images to visualize nighttime lights, specifically anthropogenically derived light, including electric lighting and fires, which are direct indicators of human presence^24^,^25^, and detect cloud cover daily. We used DMSP-OLS images from the F15 satellite exclusively, which was launched in 2000 and provided usable night lights images until 2008^34^. The spatial resolution of DMSP OLS daily captured imagery is ~1 km, permitting analyses of brightness within cities. Brightly lit areas can cause sensor saturation while low-lit areas may be undetectable^35^.

Images are screened for cloud cover and light contaminants, such as non-anthropogenic sources of illumination, which can impact brightness measurements. We screened all TIR images for cloud presence over all pixels for all areas of interest visually and with an automated extraction using the ArcGIS spatial analysis package. A conservative threshold was chosen (50 in digital number (DN) format, consistent with^17^ and others), and images with TIR values above this threshold were not used. To avoid solar contamination and reduce the impact of variability in human behavior, we only screened images that were captured between 7 pm and 10 pm and did not use images captured during bright moon phases.

155 images were used in this study, all were captured in 2000, 2002, 2003, and 2004; 2001 was not used due to clouds. Satellites drift and sensors gradually degrade over time, so brightness values were calibrated for comparability across years using intercalibration equations (see Table 2 in ref. 34). To create an annual signature of brightness for each city, commune, and quartier, we arranged the intercalibrated images by Julian date.

We calculated the mean brightness within each city, commune, or quartier for each image. City-level brightness values were used for correlation against city-wide estimated measles transmission rates (Fig. S1, adapted from ref. 17). A cubic smoothing spline (df = 3) was fit to the relative brightness values for visualizations are shown as smoothed curves for each city and commune (Figs 1C, 2 and S1).

### Modeling population size fluctuations

We previously fit an SEIR model with migration to estimate the relationship between change in brightness and the susceptible population, due to both immigration and emigration^17^. We build upon that model and calculate the total population size throughout seasonal fluctuations. For each commune, we estimate time dependent migration (M_t_) into and out of the susceptible (S), exposed (E) and recovered (R) classes:


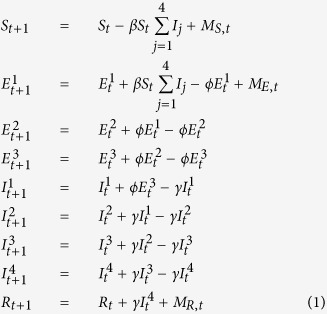


S_t_, E_t_, I_t_ and R_t_ are the number of susceptible, exposed, infectious, and recovered individuals at time *t*, respectively, and β is a constant transmission parameter. Infected individuals progress through the stages of the exposed and infectious classes at rates *ϕ* = 1/2 and *γ* = 1/2 to approximate gamma distributed durations in each class of 6 and 8 days, respectively^36^,^37^. The reinforcement activity was modeled as a reduction of the susceptible population by a total of 50%, based on the LQAS findings, at a constant daily rate, over the 7 days of the campaign.

M_S,t_, M_E,t_, and M_R,t_ represent the migrating susceptible, exposed, and recovered (i.e. immune) individuals of each commune in each time step. We assume that symptomatic individuals do not move, though the results are not qualitatively different if we allow for infectious migrants. Numbers of migrants are modeled as a linear function of the change in nighttime light brightness with slope Θ, giving the time specific changes:


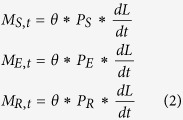


where 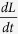 is the derivative of a smoothing spline fit to the brightness values of each commune. We assessed that 0.95 of migrants were immune (i.e. P_R_ = 0.95), consistent with the equilibrium proportion immune for a pathogen with R_0_ ≈ 20. Scaling constants P_S_ and P_E_ reflect the assumption that non-immune migrants should be disproportionately susceptible relative to the exposed classes at the ratio of P_S_:P_E_ of 50:1, a sensitivity analysis and the results from other ratios (1:1, 10:1, 25:1, and 100:1) are discussed in detail elsewhere^17^. The number of individuals transitioning between all classes (S, E, I, R) daily was assumed to be a Poisson random variable with expectation equal to the values given by equations 1 and 2.

Local health centers provided daily records of measles cases that presented at clinics at the commune level from calendar day 307 of 2003 through day 172 of 2004; cases are likely underreported. We model cases as a negative binomial random variable with mean equal to 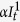 and variance equal to σ; the cases presenting at clinics each day are those transitioning from the exposed to infectious class, when symptoms of infection became apparent.

We used a Bayesian particle filter to fit the dynamic epidemic model (equations 1 and 2) to the commune level reported measles cases^38^ with additional details in ref. 17. The algorithm was:

1. 50000 draws for the parameters: β, Θ, S_0_ (the initial susceptible population size), α, and σ from uniform prior distributions.
2. For each parameter draw from the prior, 5000 iterations of the stochastic epidemic model (equations 1 and 2) were run starting with the first reported measles case in Niamey (day 307 of 2003), primed with a single infected individual.
3. For each simulated trajectory, we evaluated the likelihood of observing the reported cases given the simulated incidence, 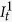 under the negative binomial observation model.
4. We calculated the approximate likelihood for each draw from the prior as the average of the likelihoods for the 5000 simulated trajectories.
5. Then we re-sampled, with replacement, the initial draws from the prior distribution with sampling weights equal to the approximate likelihood to arrive at a posterior distribution for the parameters.

The mean of the posterior distribution was our point estimate with quantiles as interval estimates.

We estimated the city’s total seasonal migrant influx at the time of the intervention as the cumulative sum of Θ*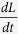, from the trough of brightness to the first day of the vaccination intervention; the mean and credible intervals of migrant influx were calculated using the mean, 2.5^th^ and 97.5^th^ quantiles of the posterior distribution of Θ. All modeling was done using R^42^.

## Results

### Vaccination campaign: population sizes and coverage estimates

**Figure 3:**
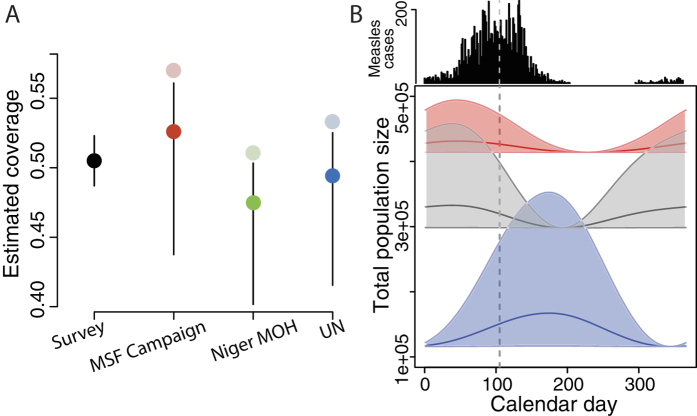
Population fluctuations and vaccine coverage . (**A**) Point estimates of coverage of reinforcement activities in the city from LQAS responses (left, black) and as calculated from doses distributed and city population size estimates from MSF, MoH, and the UN (left to right, faded points). Bright points and CI: Estimated coverage of reinforcement activities with CI including model estimates from posterior distribution of population flux using city population size estimates (bright points and lines). (**B**) Above: reported daily measles cases in Niamey. Below: estimated population fluxes of each commune by calendar day calculated from model. Vertical dashed line indicates start of reactive immunization campaign. Central solid lines indicate estimates for population flux based on posterior mean; shaded polygons indicate prediction intervals for flux based on central 95% of posterior distribution. Commune colors as in Fig. 1.

Table 2 shows the LQAS results on number and percent of children who were vaccinated before the campaign in Niamey, as well as the commune breakdowns for card verified and combined card verified and recall responses. Table 3 shows the same information for children who were vaccinated during the campaign (Fig. 3A).

The LQAS showed that the proportion of children surveyed who received measles immunization during the reinforcement activities was significantly different across all pair-wise commune comparisons (Tukey test, p < 0.05 across all pairwise comparisons for vaccine card-verified responses and for card verified + recall).

Vaccination during the campaign was not significantly associated with measles vaccination status prior to the campaign in 60 of the 64 quartiers of the city by an odds ratio test. In quartier 3 (commune 1) and quartiers 24 and 37 (commune 2), children with prior vaccination were more likely to receive a vaccination during the campaign (p < 0.05) and in quartier 27 (commune 2), previously unvaccinated children were more likely to be vaccinated during the campaign (p < 0.05). The results were consistent when using only card verified responses as well as all responses (card-verified + recall).

The largest population size estimate for Niamey in 2004 came from MOH and suggested a target population size that was 17000 children greater than *MSF*’s estimate. Simply using this population size and the number of immunization doses delivered, we calculated 51% coverage by the campaign. Using the UN estimate for the same calculation reveals 53% coverage. Both of these estimates were closer to the 50.5% coverage that was measured by the LQAS than *MSF’s* 57% estimate (Fig. 3A).

### Seasonal migration and population sizes: modeling the population and the outbreak

In addition to uncertainty in the resident population size of the city of Niamey in 2004, the immunization campaign occurred during the dry season, when the city’s population size had increased with seasonal migrants^17^,^26^. None of the population size estimates included this temporary increase but it likely contributed to the difference between the LQAS’s measurements of campaign coverage and the other estimates (Fig. 3).

Our dynamic disease model uses satellite-derived brightness measurements and measles case records to quantify the seasonal population fluxes of the entire city as well as for each commune. The model conservatively estimated that, at the time of the reinforcement vaccination, the city’s total population size was 8.3% larger than its annual minimum (posterior mean for population flux; central 95% of posterior distribution 0.1–39%; individual commune fluxes shown in Fig. 3B) (see SI) but smaller than the peak population size. In absolute numbers, this means the resident target population size for the city was likely underestimated by over 11,000 children under five years of age who were present in the city when the vaccination campaign was implemented (Fig. 3B). Increasing the *MSF* target population estimate by 8.3% to account for migration indicates that the number of doses delivered would have actually achieved 52.3% (CI 45.9–56.9) coverage in the city of Niamey. Doing the same for the other two estimates of population size with an 8.3% increase for migration, we estimate 47.5% (CI 40.2–51.1) and 49.4% (CI 42.0–53.0) coverage achieved for the MoH and UN population sizes, respectively.

The UN estimate with brightness-derived migration produces the closest coverage estimate to the LQAS measurement.

### Model estimates of population fluctuations and the impact of vaccination timing

We explored potential changes in coverage by varying the date of onset of a reactive vaccination campaign. We modeled immunization campaigns that began on each day, starting from the trough of brightness to the peak in measles cases; note that the earliest of these interventions would have occurred prior to the onset of the 2003–2004 outbreak. We simulated measles outbreaks from the first reported case of the outbreak and modeled each vaccination campaign to last about two weeks and to target 90% of the total (resident and migrant) population that would be present at the time of the campaign. Although the 2004 campaign targeted only 50% coverage, we modeled 90% successfully immunized because this level of coverage would approach measles herd immunity. While 95% population level immunity is preferable, older age groups in a population with recurring outbreaks should have high levels of natural immunity. For each simulation, the population influx into the city was modeled as the mean of the posterior estimate of Θ*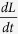.

**Figure 4:**
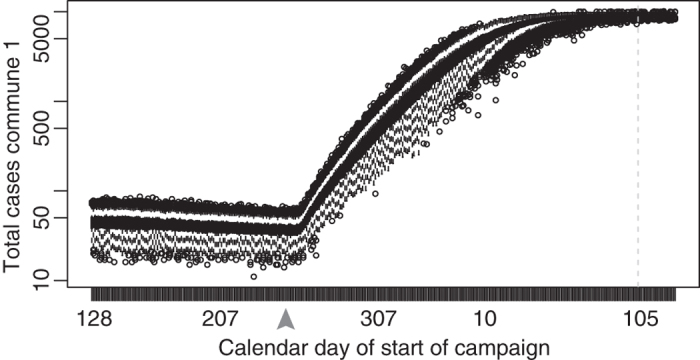
Vaccination time and outbreak size, commune 1, Niamey. Boxplots of the predicted total size (central 90%) resulting from a measles outbreak if a two-week campaign vaccinating 90% of the population began on the day of the x-axis. The outbreak started on day 307, 2003; the immunization campaign began on day 105 of 2004, shown by the dashed vertical line. A campaign beginning as late as ~day 230 (grey arrow) can theoretically result in a smaller total outbreak size but best practice remains to vaccinate early in response to an outbreak.

Although the influx of susceptible migrants occurs over several months, the simulations show that, in response to an outbreak, an early intervention consistently results in fewer cases than a later intervention (commune 1 Fig. 4, commune 2 Fig. S2). There is a brief period around day 230, immediately before the total number of cases begins rising rapidly, when the model predictions suggest that a precisely timed campaign in commune 1 would result in fewer total measles cases than an earlier campaign by immunizing a greater total number of incoming susceptible migrants (Fig. 4). However, the benefit is trivial and the risk of missing this narrow window for optimal vaccination coverage results in hundreds of preventable cases or more and the optimal window can only be definitively identified retrospectively. The simpler recommendation to vaccinate as early as possible following the detection of an outbreak remains a sound strategy.

### Extended applications: Nighttime lights from Maradi and Zinder

Nighttime brightness values show fluctuations for the cities of Maradi and Zinder, which are strongly, positively correlated with each city’s seasonal measles outbreaks (Fig. S1B,C). Despite an absence of commune-level measles case reports or vaccination coverage estimates for the second and third largest cities in Niger, the fluctuations in brightness values provide relatively high spatiotemporal resolution measurements for the fluctuations in city and commune population sizes (Fig. 2A,B). This would be useful in estimating the target population size for vaccination campaigns and other public health efforts.

## Discussion

We consider the LQAS measurement of 50.5% (Fig. 3A) to be the most accurate reflection of the vaccine coverage achieved by the immunization campaign. In the absence of the LQAS, measles vaccine coverage in Niamey following the 2004 campaign would have been overestimated. This would play an important role in future vaccination goals and immunization dose distribution.

Inaccurate population size estimates and a lack of planning for migration underlie low vaccination coverage and unexpected, preventable disease outbreaks. Despite routine immunizations and repeated catch-up and supplementary campaigns, many countries continue to experience ongoing measles epidemics. In Niger specifically, recent estimates of vaccination coverage in children younger than one suggest local elimination of measles^39^ but these are likely overestimates, as outbreaks continue to occur.

In areas with high routine immunization coverage in residents and predictable, reccurring population fluctuations, *pre-planned* supplemental vaccination campaigns can be implemented near the peak in population size if outbreak risk is very low. This strategy provides comprehensive coverage to migrant populations, who may be otherwise difficult to reach. However, while timing a *preventative* intervention to coincide with a large population presence can increase the reach and coverage of a campaign, our model simulations warn against targeting *reactive* vaccinations to coincide with seasonal maximums in population sizes (Fig. 4)^31^. For reactive vaccinations, the conventional wisdom of responding as quickly as possible shows the greatest benefit, regardless of population fluctuations. In cases where increases in population size lead to elevated contact rates and increased disease transmission, reactive vaccination campaigns will benefit greatly from estimates of seasonal or short-term migrant populations. Of course, all vaccination strategies will improve coverage and estimates by accurately calculating the size and distribution of the target population.

Satellite imagery enhances our abilities to remotely estimate population sizes, assess fluctuations, and update public health as local populations shift. Even without detailed measles case records and immunization coverage for Maradi and Zinder, satellite images show city-level seasonal patterns of population fluctuations that correlated measles incidence and commune-level fluctuations that showed some variation (Fig. S1B,C). This makes it possible to improve retrospective estimates of prior vaccination campaigns and increase the coverage of future campaigns with accurate calculations of doses and timing (SI, Fig. 2). Additional public health programs can use these methods to increase accessibility to care or services.

The serial satellite imagery used here is publicly accessible. Even higher resolution nighttime lights images captured daily after 2012 by Suomi NPP VIIRS are freely available^40^. Movement can also be measured using detailed individual mobility traces extracted from privately-owned, anonymized mobile phone call detail records (CDR)^41^. Unfortunately, currently, CDRs may reflect local biases in usership and service coverage and carry significant privacy issues, which require legally binding non-disclosure agreements^42^. These issues make data-sharing with collaborators and aid-providers difficult but will hopefully improve as CDRs become increasingly pervasive and actionable^15^,^43^.

Satellite-derived measurements of population fluctuations can improve the logistics of vaccination campaigns and public health interventions by improving estimates of the necessary number of vaccine doses and the resources required for distribution, particularly in agricultural economies with seasonal migration. In addition to critically evaluating estimates of population sizes, public health intervention strategies can improve their reach and coverage and increase their impact by accounting for migration in the two ways detailed below.

First, current immunizations and intervention strategies that occur at regular intervals and target specific age groups use annual population estimates, which overlook recurring or cyclic patterns of mobility. This can repeatedly miss a segment of the population, resulting in underestimates of the number of susceptible individuals remaining in the population. These susceptibles can accumulate silently over time, priming conditions for large, unexpected outbreaks. By quantifying population fluxes, it is possible to assess the *uncertainty* around population size estimates, and therefore the uncertainty in immunization coverage estimates, based on spatiotemporal variance in brightness patterns.

Second, opportunistically planning non-reactive vaccination campaigns to deliberately coincide with increases in population size can improve the reach and accessibility of intervention efforts by including seasonally migrating individuals when they are in urban areas with relative ease and low cost. Many migrant groups can be otherwise difficult to reach in rural or remote areas^15^ though if an outbreak is likely to be triggered by an increase in population size, high-coverage vaccination efforts concentrated during seasonal troughs in population size may be effective in large outbreak prevention.

While the importance of comprehensive post-campaign surveys cannot be stressed enough, in cases where they absolutely cannot be implemented, including for retrospective estimates of previous campaigns, the method presented here can improve estimates of coverage and uncertainty for immunization campaigns.

### Conclusions

Gaining an understanding of local population sizes, movement, and migration patterns helps make informed changes in the planning of an immunization campaign in a data-rich location like Niamey. Changes in population size can be remotely measured with widely available satellite imagery of anthropogenic lights, and can be applied to vaccinations and other pubic health efforts in areas without detailed disease or immunization data, as demonstrated here for Maradi and Zinder in Niger. Additional applications include other infectious diseases, including, poliovirus, meningococcal meningitis, and vector control strategies. Polio remains endemic in two countries^44^ with control measures often targeting the movement of infected and susceptible individuals^6^. Meningococcal meningitis in Sub-Saharan Africa displays seasonal fluctuations and spatial patterns similar to measles in the same region^45^,^46^. Vector control campaigns that reach more people, including urban residents and migrants, will have a greater impact on regional control of malaria and other vector-borne pathogens, including the recent outbreak of Zika virus. Accurate measures of population movements can broadly improve immunization, disease surveillance, drug and prophylactic distribution, and basic health care and have the potential to transform control strategies for transmissible diseases in the regions where improvement is most desperately needed and has been most difficult to achieve.

## Additional Information

**How to cite this article**: Bharti, N. *et al*. Measuring populations to improve vaccination coverage. *Sci. Rep.* **6**, 34541; doi: 10.1038/srep34541 (2016).

## Supplementary Material

Supplementary Information

## Figures and Tables

**Table 1:** Summary of demographic data.

Data source	MSF	MoH	UN
Base pop size	2001 census; 707951^30^	2001 census; 707951	2001 census; 727000
Growth rate	annual 4.8%	annual 4.5%	2002: 3.9%; 2003: 2.9%; 2004: 2.85%
Pop under 5	19.30%^48^	21%	20%^49^
Est target pop size	148600	168769^30^	158600
Est 2004 pop size	769454	807890	793000^50^

**Table 2:** LQAS results for children who were vaccinated before the reinforcement.

	City# vax	City% vax	C1% vax	C2% vax	C3% vax
Card verified (n = 2348)	1786	76	71	87	74
Card + recall (n = 2987)	2129	71	68	78	66

City total; city percent; commune 1 percent; commune 2 percent; commune 3 percent.

**Table 3:** LQAS results for children who were vaccinated during the reinforcement.

	City# vax	City% vax	C1% vax	C2% vax	C3% vax
Card verified (n = 1813)	1055	58	46	58	74
Card + recall (n = 2988)	1511	51	41	50	71

Columns same as Table 2.
