# Supplementary material for: Measuring populations to improve vaccination coverage
Source: Sci Rep. 2016 Oct 5;5:34541. doi: 10.1038/srep34541 (PMC5050518; doi:10.1038/srep34541)
Supplement: Supplementary Information [file srep34541-s1.pdf]

Measuring populations to improve vaccination coverage

Nita Bharti[1,2*], Ali Djibo[3], Andrew J. Tatem[4,5], Bryan T. Grenfell[4,6], Matthew J. Ferrari[1,7]

[1]Biology Department; Center for Infectious Disease Dynamics, Pennsylvania State University, University Park PA
[2]Woods Institute for the Environment, Stanford University, Stanford CA, visiting scholar
[3]Université de Niamey, Niamey, Niger
[4]Fogarty International Center, National Institutes of Health, Bethesda , MD
[5]Department of Geography, University of Southampton, Southampton, UK and Flowminder Foundation, Stockholm Sweden
[6]Department of Ecology and Evolutionary Biology; Woodrow Wilson School of Public and International Affairs, Princeton University, Princeton NJ
[7]Department of Statistics, Pennsylvania State University, University Park PA
[*]Correspondence to: nita@psu.edu

Supplementary Information: Details of vaccination campaign, LQAS, and model.

Migration and reinforcement immunization coverage

To assess the impact of migration on vaccination coverage in Niamey during the 2004 immunization campaign, we compared commune and quartier levels of the coverage achieved during the reinforcement activities to the commune- and quartier-level changes in anthropogenic brightness, as measured from satellite imagery. We considered vaccine card-verified responses as well as responses that were based on recall and could not be card-verified. We measured the brightness and population density at the time of the reinforcement activities for the formal commune boundaries and their associated peripheral areas: communes 1 and 2 were decreasing, having passed their peaks for brightness and measles cases, while commune 3 was increasing in both population size and measles cases during the reinforcement activities (Fig. 1B inset, all points; Fig. 1C dashed lines).

The brightness curves for the three communes of Niamey, when looking within the official commune boundaries only, showed that the seasonal population fluctuations of commune 2 were relatively flat and peaked later than commune 1, while commune 3 peaked last (Fig. 1B inset, bright points only; Fig. 1C, solid lines). At the time of the reinforcement activities, 78.5% of the quartiers of commune 2 and 100% of the quartiers of commune 3 were increasing in brightness and population density while 80% of the quartiers of commune 1 were past their peak in brightness and were decreasing in population density (Fig. 1C and Fig S3).

From the LQAS results, we found a positive correlation between the proportion of children who were vaccinated (with card verification) in each quartier during the reinforcement activities and the change in brightness by quartier at the time of the reinforcement activities (corr = 0.62; CI 0.36-0.78)(Fig. S3). We also found that the total number of children (card and recall) vaccinated in each lot during the reinforcement activities was positively correlated with the change in brightness by quartier (corr = 0.42). Spatial autocorrelation was low for survey-based quartier-level immunization coverage during the reinforcement (Moran's I = 0.17) but was higher for quartier-level

mean brightness (Moran's I = 0.61) and quartier-level change in brightness at the time of the vaccination (Moran's I = 0.65).

Although the campaign had uniform coverage goals for the entire city, the results of the LQAS and the quantified brightness levels indicated that the reinforcement activities achieved higher vaccination coverage in quartiers where the population size was increasing at the time of the vaccination campaign when compared to the quartiers where population size was decreasing at the time of the vaccination campaign (Fig. S3). Importantly, there was no correlation between the campaign-achieved quartier-levels of vaccine coverage and mean brightness (corr = 0.18) or brightness variance (corr = -0.08), indicating that neither average population size nor variance in population size impacted the campaign's coverage. Instead, the change in the population size of each quartier at the time of the campaign captured an element of the population dynamics that was correlated to the campaign's coverage. The mechanism underlying this pattern is unknown; it may suggest that the migration cycle itself, not just the absolute numbers of people, might be important in predicting the uptake of vaccination.

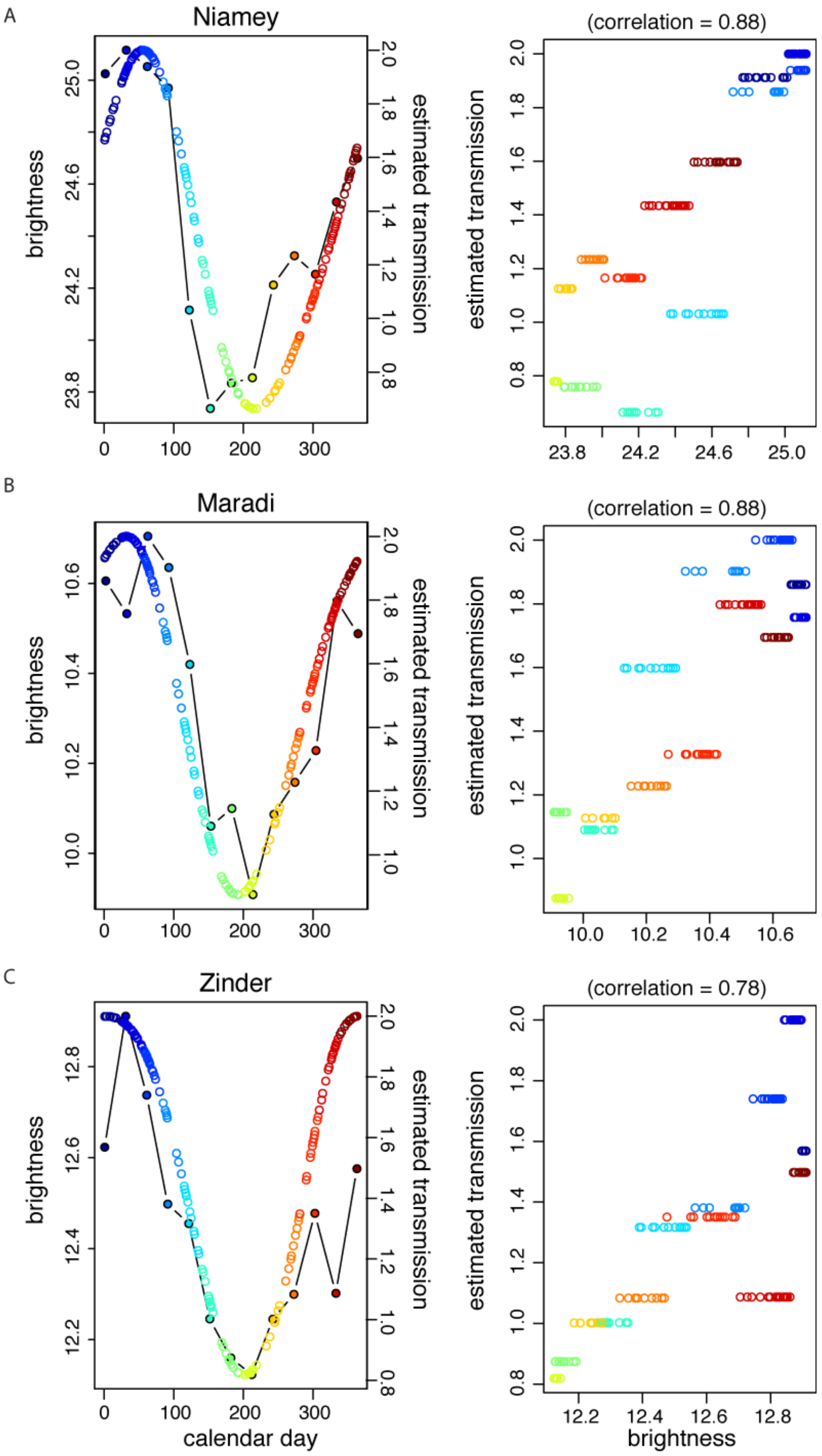
A
Niamey
(correlation = 0.88)
B
Maradi
(correlation = 0.88)
C
Zinder
(correlation = 0.78)
brightness
estimated transmission
calendar day

Fig. S1 Brightness and measles transmission. (A) Niamey. Left: annual brightness pattern against day of year shown in open circles that progress along the spectrum from blue to red corresponding to time of year (January to December); estimated measles transmission rates for biweekly time steps shown in circles connected by black lines. Right: estimated transmission rates against brightness values; colors correspond to time of year as on left. (B) Same as (A) for Maradi. (C) Same as (A) for Zinder.

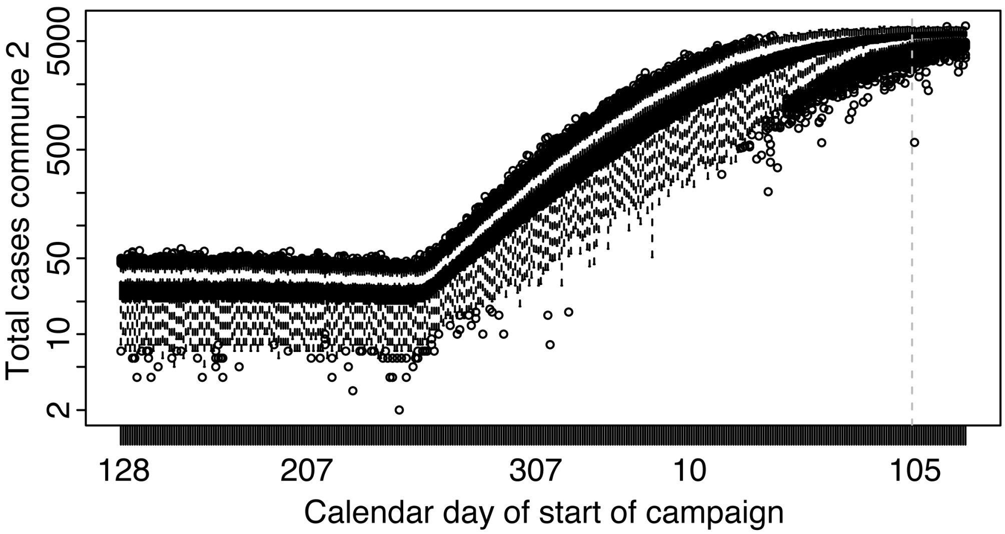

Fig. S2 Vaccination time and outbreak size, commune 2, Niamey. Boxplots of the predicted total size (central 90%) resulting from a measles outbreak if a two-week campaign vaccinating 90% of the population began on the day of the x-axis. The outbreak started on day 307, 2003; the immunization campaign began on day 105 of 2004, shown by the dashed vertical line. The best practice is to vaccinate early in response to an outbreak.

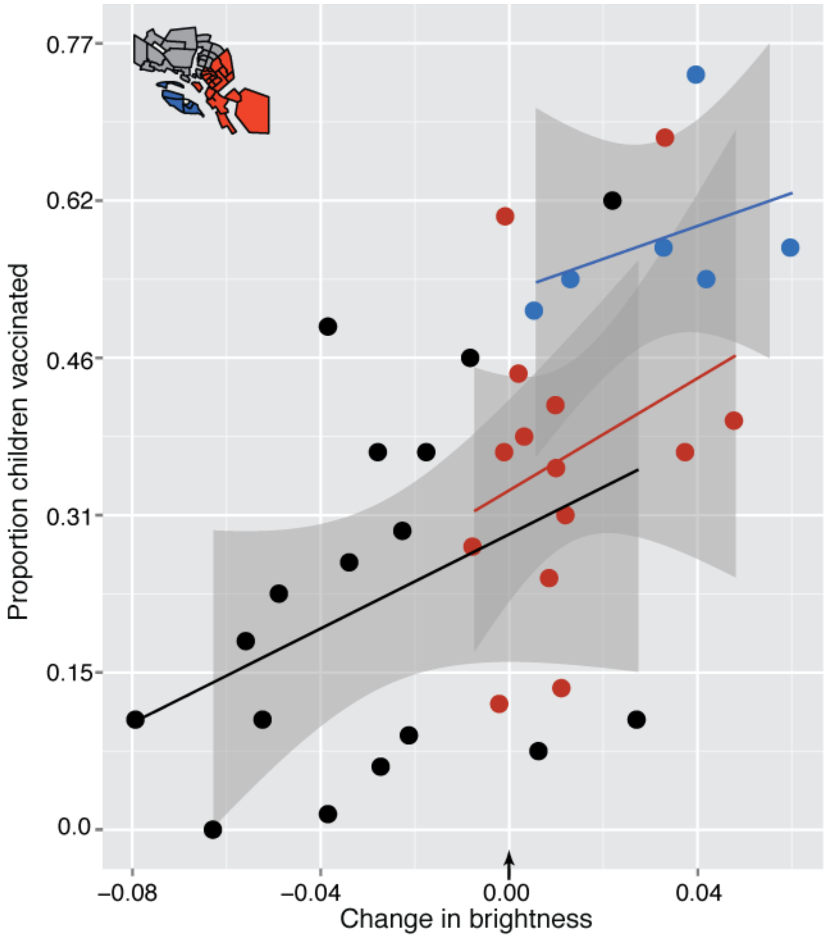

Fig. S3 Brightness and measles immunization. Proportion of children vaccinated in each quartier during reinforcement activities from LQAS against the change in brightness of each quartier during reinforcement activities. Shaded polygons give the 95% CI for mean proportion vaccinated for each commune. Arrow on x-axis indicates zero change in brightness; quartiers to the left were decreasing during vaccination campaign, quartiers to the right were increasing. Inset map shows all quartiers. Inset map was created from data provided by *Médecins Sans Frontieres* and Niger Ministry of Health, generated in ArcGIS (version 10.0; https://www.arcgis.com/features/), drawn and edited in R version 1.7.1 (https://cran.r-project.org), finished in Adobe Illustrator CS3 (http://www.adobe.com/products/illustrator.html).
